# Supplementary material for: Lipocalin 2 Deficiency Restrains Aging-Related Reshaping of Gut Microbiota Structure and Metabolism
Source: Biomolecules. 2021 Aug 28;11(9):1286. doi: 10.3390/biom11091286 (PMC8466870; doi:10.3390/biom11091286)
Supplement: Supplementary file 1 [file biomolecules-11-01286-s001.zip › biomolecules-1358308-supplementary.pdf]

Figure S1

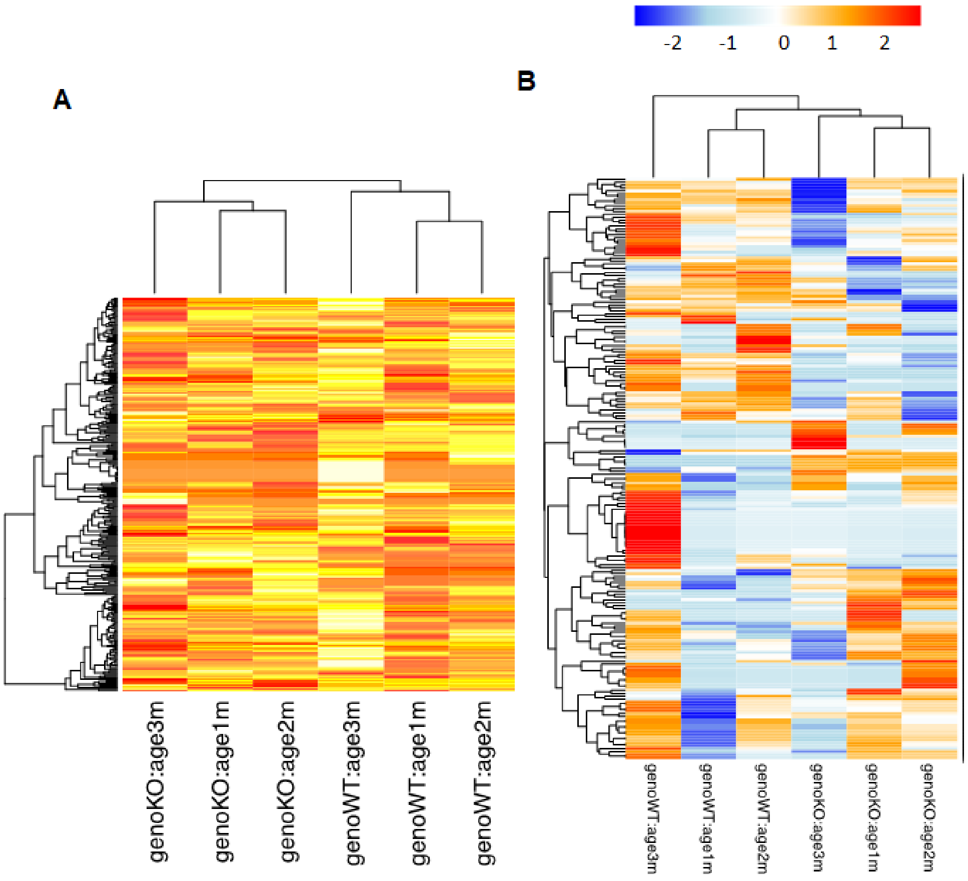

Figure S1: Age-related gut microbiota at the ASV level in WT and LKO mice. (**A**) HCA-based heatmaps on 241 ASVs that have variance > 1e-06. (**B**) HCA-based heatmaps on differentially abundant ASVs between WT and LKO mice. The increased abundances of 67 ASVs and decreased abundances of 132 ASVs were observed in LKO mice. The inputs for creating heatmap are derived from a negative binomial generalized linear model in the R environment. n = 6-9. q value ≤ 0.05 and log2 scale fold change ≥ 2 are the criteria to determine the significance.

Figure S2

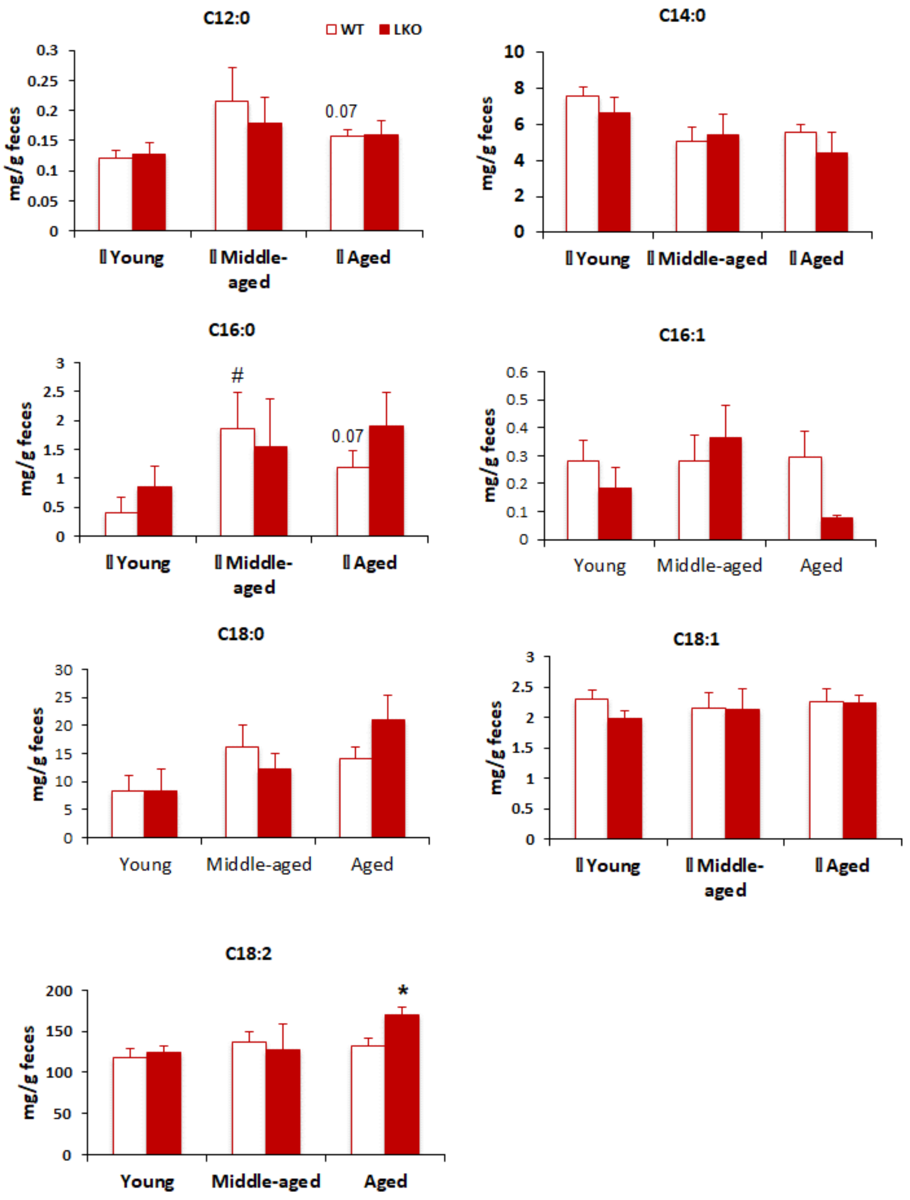

Figure S2: Fecal LCFA levels in WT and LKO mice. Stool samples from WT and LKO at the age of 6 months (young), 12 months (middle-aged) and 18 months (old) were homogenized in 50% ACN. Supernatants were collected for HQ reaction to detect fecal LCFA levels. Data are presented as mean ± SEM (n = 6-9). # $P < 0.05$, ## $P < 0.01$ versus young WT mice.

Table S1: Real-time qPCR primer sequences

Target	Forward primers (5′ to 3′)	Reverse primers (5′ to 3′)
TNFα	CAGAAACACAAGATGCTGGG	CAAAAGAGGAGGCAACAAGG
IL-6	AGAAGGAGTGGCTAAGGACCAA	AACGCACTAGGTTTGCCGAG
IFNγ	TGAACGCTACACACTGCATCT	TGTCACCATCCTTTTGCCAGT
IL-17	AAACGTGGGGGTTTCTTAGG	TTCAGGGTCGAGAAGATGCT
Arg1	AACACGGCAGTGGCTTTAACC	GGTTTTCATCTGGCGCATTC
IL-10	CATGGCCCAGAAATCAAGGA	GGAGAAATCGATGACAGCGC
FFAR2	CACAGGAAACGGGAAGCCTCG	CTGGGGTCATTCTCCTTGGGC
FFAR3	CAGCAGAGTGCCAGTTGTCCA	GAAGACCACCAGGGCCATCAC
Tbp2	GAAGAACAATCCAGACTAGCAGCA	CCTTATAGGGAACTTCACATCACAG
